# Prevalence and associated factors of COVID-19 among biomedical science students of Rivers State University, Port Harcourt, Nigeria: a cross-sectional study

**DOI:** 10.1097/j.pbj.0000000000000283

**Published:** 2025-03-18

**Authors:** Moore I. Mike-Ogburia, Gift M. Hart, Barynem Vito-Peter, Aisha Dio, Victory C. Nwogu

**Affiliations:** a Department of Medical Laboratory Science, Rivers State University, Port Harcourt, Nigeria; b School of Public Health, University of Port Harcourt, Port Harcourt, Nigeria

**Keywords:** COVID-19, SARS-CoV-2, associated factors, biomedical science, medical laboratory science, Rivers State University

## Abstract

**Background:**

The coronavirus disease 2019 (COVID-19) pandemic has profoundly affected global health, emphasizing the need to assess specific populations such as university students to better understand the prevalence of the infection and its determinants. This study investigated the prevalence and associated factors of COVID-19 among biomedical science students at Rivers State University, Port Harcourt, Nigeria.

**Methods:**

A cross-sectional study was conducted among 220 students from March to August 2022. Data collection was performed using interviewer-administered questionnaires while COVID-19 infection was screened using the Panbio™ COVID-19 Ag Rapid Test Device. Data analysis involved both descriptive and inferential statistics, with statistical significance set at *P* ≤ .05.

**Results:**

The prevalence of COVID-19 was 11.4% (95% CI: 7.8–16.2). Significant associations were observed between COVID-19 prevalence and facemask use, handwashing practices, use of sanitizers, and nature of accommodation. However, in the multivariable analysis, only noncompliance with facemask use was identified as a significant independent predictor of COVID-19 infection (aOR = 4.350, 95% CI: 1.379–14.13, *P* = .0124).

**Conclusion:**

The study highlights a concerning prevalence of COVID-19 among biomedical science students. Tailored public health strategies are essential, emphasizing compliance with preventive measures as a means to mitigate COVID-19 transmission within this crucial demographic.

## Abstract 2

Supplemental Digital Content is Available in the Text.

## Introduction

The outbreak of the novel coronavirus disease 2019 (COVID-19) has had an unprecedented impact on global health, societal well-being, health care systems, and resources.^1,2^ As the global scientific community strives to combat the virus, it is imperative to investigate specific populations to shed light on the prevalence of the infection within such populations and predisposing factors to the infection.

Young adults, including university students, represent a significant demographic regarding both susceptibility to the virus and potential for transmitting it within communities.^3^ Biomedical science students, with their strong scientific backgrounds, are uniquely positioned to contribute to the understanding and management of COVID-19. Their perceptions of the disease and adherence to recommended safety and preventive measures could play a vital role in shaping public health outcomes within and beyond the university community.

Rivers State University, located in Port Harcourt, Nigeria, inhabits a microcosm of young, aspiring biomedical professionals who will play an important role in shaping the future of health care in their region. However, limited research has been conducted to evaluate the prevalence of COVID-19 and its associated risk factors specifically among this cohort in Nigeria. Understanding the dynamics of COVID-19 epidemiology in this context is crucial for tailoring educational campaigns, promoting COVID-19 preventive measures, and effectively addressing any barriers that may exist particularly as students are at a higher risk of acquiring and transmitting the virus, under the false assumption of being invulnerable to the infection.^4^ Given the aforementioned, this study aimed to determine the prevalence and associated factors of COVID-19 among biomedical science students of Rivers State University, Port Harcourt.

## Materials and methods

### Study design and area

**Figure 1.**
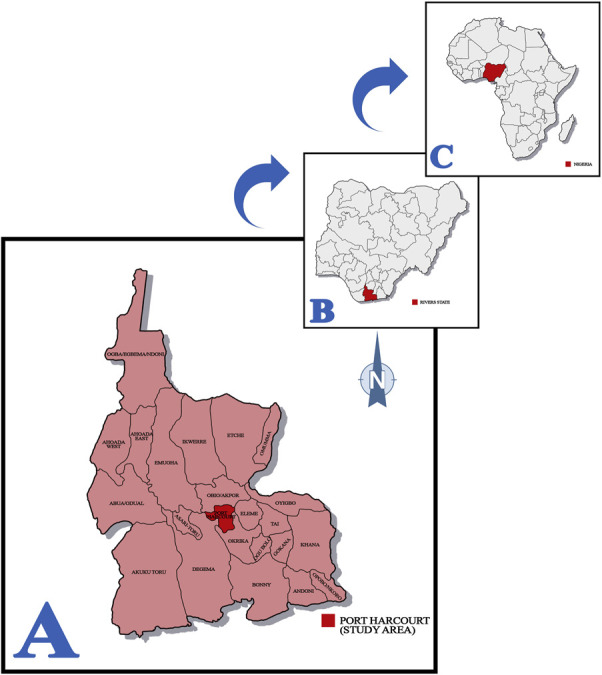
A, Map of Rivers State highlighting Port Harcourt (study area); (B) map of Nigeria highlighting Rivers State; (C) map of Africa highlighting Nigeria.^5^

A cross-sectional study design was used to investigate the prevalence of COVID-19 and associated risk factors among biomedical science students at Rivers State University, Port Harcourt, from March to August 2022. The study was specifically conducted in the Department of Medical Laboratory Science. This department serves as the educational hub for biomedical science students (commonly referred to as Medical Laboratory Science students) at Rivers State University. Port Harcourt, the capital city of Rivers State (Fig. 1),^5^ provides a dynamic backdrop for examining the epidemiology of the infection among these students within the broader societal and regional context.

### Study population, sample size, and sampling technique

The study population consisted exclusively of undergraduate students enrolled in the Department of Medical Laboratory Science. The sample size for this study was determined to be 200 students after using a sample size calculator,^6^ based on the 15.2% prevalence of COVID-19 in a previous study^7^; however, an additional 10% of the sample size was added to cater for nonresponse, bringing the sample size to a total of 220 students. A stratified sampling technique was used to ensure representation from different academic levels within the cohort. Specifically, 50 students were included from both first-year and second-year classes while each subsequent academic level (third to fifth year) comprised 40 students.

### Data collection

The data collection for this study used an interviewer-administered questionnaire (Supplemental Digital Content 1, http://links.lww.com/PBJ/A43). The questionnaire assessed the participant demographics, COVID-19 preventive practices/social contact, and general symptoms observed in the past 14 days and was developed from existing literature.^3,8,9^

Screening for COVID-19 infection was performed using the Panbio™ COVID-19 Ag Rapid Test Device (Abbott Rapid Diagnostics, USA) according to the manufacturer's standard operating procedure. This rapid antigen test device has been demonstrated to have a sensitivity of 85.5% and specificity of 100%^10^ while another study reported a sensitivity of 86.8% and specificity of 99.9%,^11^ which meets the WHO criteria of ≥80% sensitivity and ≥97% specificity.^12^ However, the Panbio COVID-19 Ag Rapid Test Device has demonstrated varying levels of sensitivity and specificity in some other studies. In a Kenyan study, its overall sensitivity was 46.6% (95% CI: 42.4–50.9%) and specificity was 98.5% (95% CI: 97.8–99.0%), with higher sensitivity in symptomatic cases (60.6%) compared with asymptomatic cases (34.7%).^13^ Another study reported a sensitivity of 64.7% (95% CI: 47.9–78.5%) and a specificity of 100% (95% CI: 97.0–100.0%) when using nasal swabs; although the sensitivity of the test improved to 81.3% in high viral load cases (Ct ≤ 30),^14^ making it a useful tool for detecting infectious cases, though confirmatory reverse transcription-polymerase chain reaction (RT-PCR) testing remains essential for lower viral load or asymptomatic cases in addition to interpreting negative results with caution.

### Data analysis

Data analysis was conducted using Graph Pad Prism 9, employing both descriptive and inferential statistics such as chi-square tests and multivariable logistic regression analyses to unveil key insights into the prevalence of COVID-19 infection among the study participants. Statistical significance was determined as a *P*-value of less than or equal to .05.

### Ethical consideration

The aim of the study was duly communicated to the students before obtaining informed consent. In addition, consent was obtained from the parents/guardians of willing students younger than 18 years before enrollment in the study. Ethical clearance was given by the Department of Medical Laboratory Science, Rivers State University (MLS/2022/ERC/UG/DE.2017/4737).

## Results

A total of 220 students aged between 16 and 32 years (mean age = 21.45 years) participated in the study. Most of the participants were between the age group of 16–20 years (45.9%) and 21–35 years (41.8%), mostly female (64.1%) and single (95.5%). Most participants were unemployed (62.7%); a total of 82 participants were part-time employees, of whom 85.4% were working in establishments unrelated to health.

**Figure 2.**
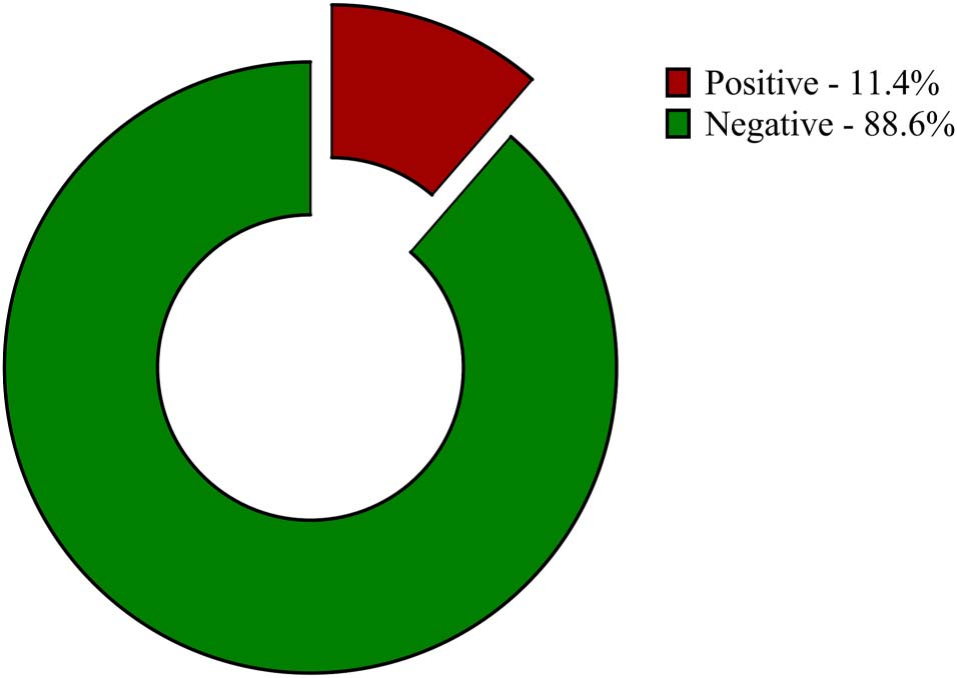
Prevalence of COVID-19 among study participants.

The prevalence of COVID-19 was 11.4% (95% CI: 7.8–16.2) (Fig. 2) with no significant associations with the sociodemographic characteristics of the respondents (*P* > .05), as presented in Table 1. Variables such as facemask use, handwashing practice, the use of sanitizers, and the nature of accommodation were significantly associated with the prevalence of COVID-19 (*P* < .05), as presented in Table 2. However, in the multivariable logistic regression analysis of factors associated with COVID-19 infection, only noncompliance with facemask use (aOR = 4.350, 95% CI: 1.379–14.13, *P* = .0124) was found to be a significant independent determinant of COVID-19 infection as presented in Table 3.

**Table 1:** Association between the prevalence of COVID-19 and the sociodemographic characteristics of study participants

Variable	N	Positive (%)	Negative (%)	χ^2^	Df	*P*
Age						
16–20	101	11 (10.9)	90 (89.1)	2.294	2	.3175
21–25	92	13 (14.1)	79 (85.9)			
≥26	27	1 (3.7)	26 (96.3)			
Total	220	25 (11.4)	195 (88.6)			
Sex						
Male	79	6 (7.59)	73 (92.4)	1.738	1	.1874
Female	141	19 (13.5)	122 (86.5)			
Total	220	25 (11.4)	195 (88.6)			
Marital status						
Single	210	25 (11.9)	185 (88.1)	1.343	1	.2465
Married	10	0 (0)	10 (100)			
Total	220	25 (11.4)	195 (88.6)			
Academic year						
Lower years	100	12 (12)	88 (88)	0.07371	1	.7860
Upper years	120	13 (10.8)	107 (89.2)			
Total	220	25 (11.4)	195 (88.6)			
Occupation (besides studies)						
Unemployed	138	16 (11.6)	122 (88.4)	0.01954	1	.8888
Employed	82	9 (11)	73 (89)			
Total	220	25 (11.4)	195 (88.6)			
Nature of employment (N = 82)						
Unrelated to health	70	7 (10)	63 (90)	0.4660	1	.4949
Related to health	12	2 (16.7)	10 (83.3)			
Total	82	9 (11)	73 (89)			

χ^2^, chi-square; Df, degree of freedom; N, sample.

**Table 2:** Association between the prevalence of COVID-19 and preventive measures/social contact of study participants

Variable	N	Positive (%)	Negative (%)	χ^2^	Df	*P*
Facemask use						
Yes	178	15 (8.4)	163 (91.6)	7.983	1	.0047*
No	42	10 (23.8)	32 (76.2)			
Total	220	25 (11.4)	195 (88.6)			
Handwashing practice						
Frequent	89	9 (10.1)	80 (89.9)	9.218	2	.0100*
Occasionally	112	14 (12.5)	98 (87.5)			
Not at all	19	2 (10.5)	17 (89.5)			
Total	220	25 (11.4)	195 (88.6)			
Use of sanitizers						
Frequent	27	2 (7.41)	25 (92.6)	9.486	2	.0087*
Occasionally	128	9 (7.03)	119 (93)			
Not at all	65	14 (21.5)	51 (78.5)			
Total	220	25 (11.4)	195 (88.6)			
COVID-19 vaccination status						
Vaccinated	18	3 (16.7)	15 (83.3)	0.5473	1	.4594
Unvaccinated	202	22 (10.9)	180 (89.1)			
Total	220	25 (11.4)	195 (88.6)			
Mode of transportation to class/school						
Walking	66	9 (13.6)	57 (86.4)	0.5604	2	.7556
Private vehicle	25	3 (12)	22 (88)			
Public vehicle	129	13 (10.1)	116 (89.9)			
Total	220	25 (11.4)	195 (88.6)			
Nature of accommodation						
Hostel	74	14 (18.9)	60 (81.1)	6.435	2	.0401*
Off-campus lodge	71	6 (8.45)	65 (91.5)			
Family house	75	5 (6.67)	70 (93.3)			
Total	220	25 (11.4)	195 (88.6)			
Number of roommates/flatmates						
≤2	65	8 (12.3)	57 (87.7)	0.08163	1	.7751
>2	155	17 (11)	138 (89)			
Total	220	25 (11.4)	195 (88.6)			
Number of coursemates in class						
<100	40	3 (7.5)	37 (92.5)	0.7246	1	.3947
≥100	180	22 (12.2)	158 (87.8)			
Total	220	25 (11.4)	195 (88.6)			
Number of coursemates on a bench						
≤4	32	1 (3.1)	31 (96.9)	2.523	1	.1122
>4	188	24 (12.8)	164 (87.2)			
Total	220	25 (11.4)	195 (88.6)			

* Statistical significance at *P* ≤ .05

χ^2^, chi-square; Df, degree of freedom; N, sample.

**Table 3:** Bivariate and multivariable logistic regression analyses of factors associated with COVID-19 infection among study participants

Variable	cOR [95% CI]	aOR [95% CI]	*P*
Age			
16–20 (ref)	0.9167 [0.3885–2.115]	1	
21–25	1.591 [0.6870–3.712]	1.874 [0.5193–7.098]	.3426
≥26	0.2708 [0.01485–1.371]	0.5152 [0.02387–4.219]	.5827
Sex			
Male (ref)	0.5278 [0.1853–1.313]	1	
Female	1.895 [0.7618–5.397]	1.635 [0.5895–5.121]	.3651
Academic year			
Lower years (ref)	1.122 [0.4817–2.596]	1	
Upper years	0.8910 [0.3851–2.076]	0.8012 [0.2275–2.656]	.7213
Occupation			
Unemployed	1.064 [0.4552–2.629]	0.7862 [0.3047–2.091]	.6205
Employed (ref)	0.9401 [0.3804–2.197]	1	
Facemask use			
Yes (ref)	0.2945 [0.1223–0.7309]	1	
No	3.396 [1.368–8.179]	4.350 [1.379–14.13]	.0124*
Handwashing practice			
Frequent (ref)	0.8086 [0.3277–1.886]	1	
Occasionally	1.260 [0.5463–2.971]	0.8923 [0.3277–2.472]	.8231
Not at all	0.9105 [0.1388–3.462]	0.3956 [0.04609–2.283]	.3359
Use of sanitizers			
Frequent (ref)	0.5913 [0.09145–2.173]	1	
Occasionally	1.357 [0.5736–3.468]	2.189 [0.4810–16.09]	.3612
Not at all	0.8916 [0.3107–2.242]	1.100 [0.1879–9.000]	.9196
COVID-19 vaccination status			
Vaccinated (ref)	1.636 [0.3591–5.461]	1	
Unvaccinated	0.6111 [0.1831–2.784]	0.6976 [0.1719–3.768]	.6386
Mode of transportation to class/school			
Walking (ref)	1.362 [0.5481–3.205]	1	
Private vehicle	1.072 [0.2403–3.430]	1.694 [0.2914–8.398]	.5289
Public vehicle	0.7378 [0.3185–1.721]	1.132 [0.3750–3.599]	.8281
Nature of accommodation			
Hostel	1.500 [0.6200–3.498]	2.215 [0.5937–9.044]	.2453
Off-campus lodge	1.333 [0.5522–3.102]	2.354 [0.5349–11.18]	.2644
Family house (ref)	0.4464 [0.1436–1.158]	1	
Number of roommates/flatmates			
≤2 (ref)	1.139 [0.4433–2.717]	1	
>2	0.8777 [0.3681–2.256]	1.288 [0.3279–5.356]	.7209
Number of coursemates in class			
<100 (ref)	0.5823 [0.1330–1.797]	1	
≥100	1.717 [0.5566–7.521]	1.577 [0.3523–8.778]	.5690
Number of coursemates on a bench			
≤4 (ref)	0.2204 [0.01212–1.106]	1	
>4	4.537 [0.9044–82.53]	3.772 [0.6191–73.76]	.2311

* Statistical significance at *P* ≤ .05

aOR, adjusted odds ratio; CI, confidence interval; cOR, crude odds ratio; ref, reference value.

**Figure 3.**
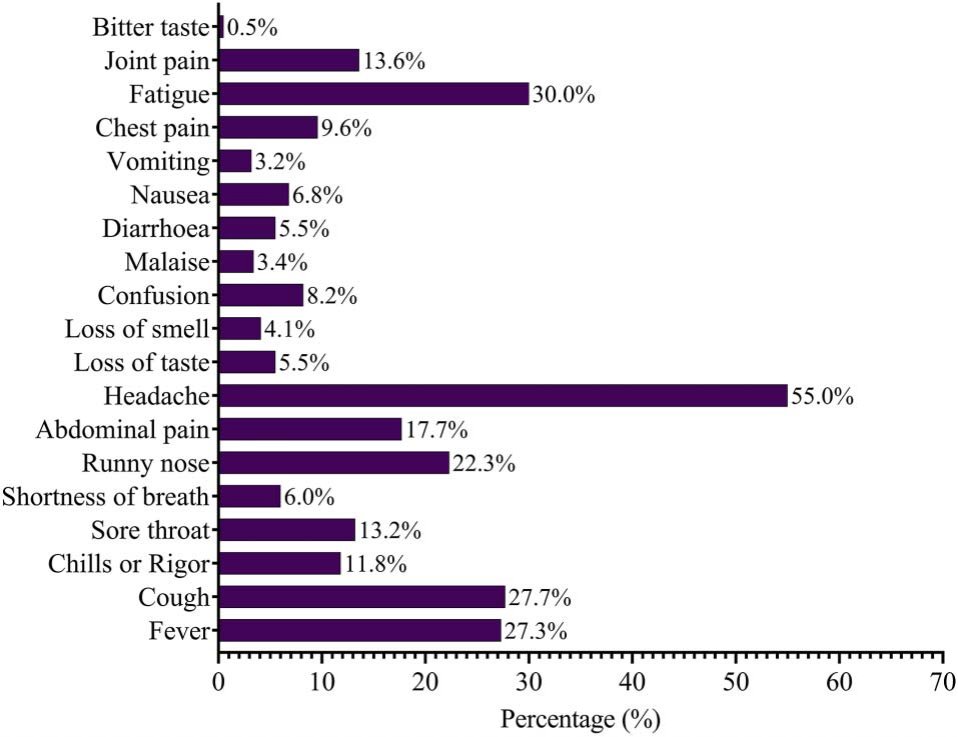
General symptoms within 14 days as reported by study participants.

The most predominant symptom in the past 14 days reported by the inmates was headache (55%), followed by fatigue (30%), cough (27.7%), fever (27.3%), and runny nose (22.3%). Chest pain was also reported by a few participants (9.6%) while only a handful reported some of the classical symptoms of COVID-19 infection such as shortness of breath (6%), loss of taste (5.5%), and loss of smell (4.1%), as shown in Figure 3.

## Discussion

The observed prevalence of COVID-19 among the students was 11.4%. This suggests that a notable proportion of students have been infected by the virus, highlighting the vulnerability of young adults, including university students, to the virus; this finding could be attributed to the close proximity and interactions of students within university campuses, particularly in settings such as classrooms, dormitories, and social gatherings. In addition, students may engage in activities outside campus that increase their risk of exposure, such as commuting to and from school, participating in extracurricular activities, or attending social events. This observation was at variance with a previous study in Port Harcourt, where no cases of COVID-19 infection were recorded among returning students of the University of Port Harcourt,^15^ and significantly lower than the prevalence reported among students in a Thai University^16^ but is, however, comparable with the findings in a study among medical students from the University of Jordan.^8^

The study revealed a significant association between noncompliance with preventive practices, such as not using facemasks, irregular handwashing, and neglecting sanitizer use, and a higher prevalence of COVID-19 among biomedical science students. This highlights the critical importance of consistent adherence to these preventive measures in mitigating the risk of infection within educational settings. Specifically, the failure to use facemasks emerged as an independent predictor of COVID-19, emphasizing its pivotal role in curbing the spread of the virus. These findings resonate with previous research, as evidenced by the study conducted by Ranjan et al,^9^ highlighting the consistent impact of facemask usage on reducing the transmission of respiratory infections. The observed association underscores the need for comprehensive public health campaigns aimed at promoting and reinforcing the adoption of preventive behaviors among students in educational institutions.

The analysis revealed a significant association between the nature of accommodation and the prevalence of COVID-19, with those living in hostels having a higher prevalence of the infection than those in personal apartments or family homes. This finding highlights the influence of close living arrangements and increased social contacts on the risk of COVID-19 transmission among biomedical science students. This aligns with previous research, as reported by Rozenfeld et al.^17^ However, the observed associations diverged from the findings of Ranjan et al,^9^ suggesting potential variability in the impact of living arrangements on COVID-19 transmission across different populations or contexts. This discrepancy highlights the need for context-specific interventions tailored to the unique dynamics of each educational institution, taking into account factors such as campus infrastructure and housing policies. Further research exploring the dynamics of living arrangements and social networks within university settings is warranted to inform targeted strategies for mitigating the risk of COVID-19 transmission among students.

The reported symptoms among the students in the past 14 days provide valuable insights into the clinical manifestations of potential COVID-19 cases within the study population. Notably, headache emerged as the most predominant symptom, with a significant 55% (121) of the students reporting this experience. This aligns with broader trends observed globally, where headache has been identified as a common symptom in COVID-19 cases.^18^ Fatigue followed closely at 30% (66), underlining the impact of the virus on the energy levels of those affected as corroborated by a previous study.^19^

Other frequently reported symptoms include cough, fever, and runny nose. These symptoms are consistent with the classical signs of respiratory infections, including COVID-19.^20^ The presence of these symptoms, especially when occurring in combination, raises concerns about potential viral transmission within the student community. It is noteworthy that while chest pain was reported by a smaller proportion, a few students also presented with more specific symptoms associated with COVID-19, such as shortness of breath, loss of taste, and loss of smell. These symptoms, although less prevalent, are considered hallmark indicators of COVID-19 infection^21,22^ and merit attention regarding both clinical and public health management.

One limitation of this study lies in the reliance on self-reported data, which introduces the potential for recall bias and social desirability bias, which can affect the accuracy of responses. The study's focus on a specific university setting may limit the generalizability of findings to broader populations. Furthermore, the use of the Panbio™ COVID-19 Ag Rapid Test Device for screening introduces additional limitations, as its moderate sensitivity may have resulted in false-negative cases, particularly among asymptomatic individuals or those with lower viral loads. While the test's high specificity minimizes false positives, its limited sensitivity necessitates caution in interpreting negative results, which could have led to an underestimation of the true prevalence of COVID-19 within the study population.

In conclusion, the study highlights a concerning prevalence of COVID-19 among biomedical science students, with noncompliance with facemask use being identified as a significant predictor of the infection. These findings emphasize the urgent need for targeted interventions addressing safety and COVID-19 preventive practices within the university to mitigate COVID-19 transmission within this crucial demographic.
